# Therapists’ experiences and needs with regard to providing work-focused care: a focus group study

**DOI:** 10.1186/s12891-021-04806-4

**Published:** 2021-11-02

**Authors:** Wiebke Oswald, Inez Ummels, Tamara Raaijmakers, Paul Baart, J. Bart Staal, Hendrik J. Bieleman, Maria W. G. Nijhuis - van der Sanden, Yvonne F. Heerkens, Nathan Hutting

**Affiliations:** 1 School of Organisation and Development, HAN University of Applied Sciences, Research Group Occupation & Health, P.O. Box 6960, 6503 GL Nijmegen, The Netherlands; 2 School for Allied Health, HAN University of Applied Sciences, Physical Therapy, Nijmegen, The Netherlands; 3 Centre Work Health, Amersfoort, The Netherlands; 4 School for Allied Health, HAN University of Applied Sciences, Key Factors in Physiotherapy and Allied Health Research Group, Nijmegen, the Netherlands; 5 Radboud Institute for Health Sciences, Radboud University Medical Centre, IQ Healthcare, Nijmegen, the Netherlands; 6 Saxion University of Applied Sciences, Research Group Health and Physical Activity, Enschede, The Netherlands

**Keywords:** Musculoskeletal disorders, Work-focused healthcare, Work participation, Physiotherapy, Occupational therapy, Exercise therapy

## Abstract

**Background:**

Musculoskeletal disorders (MSDs) can create a temporary or permanent disability that reduce a person’s ability to work. Physiotherapists (PTs), occupational therapists (OTs) and exercise therapists (ETs) are often involved in the early management of MSDs. There is a need for additional insights into therapists’ experiences, barriers and needs to work-focused care. Moreover, there is no evidence on how OTs and ETs address work participation. Therefore, the aim of this qualitative study was 1) to investigate how generalist PTs, OTs and ETs provide work-focused healthcare and 2) to obtain insight into their perceived barriers and needs that affect their ability to address occupational factors.

**Methods:**

An exploratory qualitative study using three focus groups. Generalist PTs, OTs and ETs were eligible to participate if they treated working patients with MSDs. A semi-structured interview guide with open-ended questions was developed. Two moderators facilitated each focus group using the interview guide, and all the groups were audio recorded. Data were analysed using inductive thematic analysis.

**Results:**

Sixteen therapists (mean age 44 years, range 25-59) participated in this study. Participants were aware of the importance of taking occupational factors into account. Whether they address occupational factors is largely dependent on the patient’s request for help. However, ETs and OTs consider it normal to ask about occupational factors during the diagnostic process, while PTs often address this in later consultations. Almost all participants were unaware of the existence of PTs, OTs or ETs who are specialised in occupational health. Moreover, almost all participants struggled with when to refer a patient to other (occupational) healthcare professionals. This study identified several needs of therapists. These included knowledge about laws and legislation and skills for identifying and addressing work-related or work-relevant complaints.

**Conclusions:**

Participants in this qualitative study were aware of the importance of taking occupational factors into account. However, how PTs, OTs and ETs address work participation and the extent to which they do so can be improved. There was a lack of knowledge about and cooperation with occupational health professionals, including PTs, OTs or ETs specialised in occupational health.

**Supplementary Information:**

The online version contains supplementary material available at 10.1186/s12891-021-04806-4.

## Background

Musculoskeletal disorders (MSDs) are a major cause of disability and social burden globally [1]. These conditions are characterised by pain and reduced physical functioning, resulting in decreased quality of life [2, 3]. Moreover, it has been estimated that MSDs are the greatest contributor to disability and lost productivity life years in working-aged people [1, 4].

MSDs can create a temporary or permanent disability that reduces a person’s ability to work [5, 6]. Moreover, the work environment and its characteristics, in addition to other risk factors, can play an important role in the development of MSDs [7]. Work-related MSDs are disorders in which work activities and conditions significantly contribute to the onset, continuation or progression of the disorder but are not necessarily the only cause of the disorder [8]. Although work-related environmental factors do not always directly cause MSDs, the symptoms can still be relevant to work (i.e. the work can influence the symptoms or the symptoms can affect the ability to work) [9].

High-quality clinical practice guidelines for care of people with MSDs consistently recommend that practitioners facilitate the continuation or resumption of work []. One obstacle to work participation by people with MSDs could be a lack of work-focused care (i.e. care where the healthcare professional takes an interest in, and accepts responsibility for, addressing obstacles to work participation in the clinical encounter) [11,12]. Work-focused conversations could include work-related topics such as discussing barriers and facilitators for work, setting work-focused treatment goals or updating relevant stakeholders [9].

Physiotherapists (PTs), occupational therapists (OTs) and exercise therapists (ETs) are often involved in the early management of MSDs and can play an important role in providing work-focused healthcare [9]. Therefore, they should be aware of the health benefits of participating in work and the importance of adopting a work-focused approach in their treatment [9]. Only a few studies investigated how PTs address work participation. This previous research has found that PTs often do not sufficiently address the ability to work and related occupational factors [13, 14]. Those studies found that PTs often lack specific knowledge about work-related factors and interventions, and they found insufficient cooperation between PTs and occupational healthcare professionals [13, 14]. No studies on how other healthcare professionals, such as OTs and ETs, address work participation are known. Previous qualitative research has looked at different professional populations. For example, one study included generalist PTs and PTs specialised in occupational health, which might have affected the study results [14]. Moreover, there is a need for additional insights into therapists’ experiences, barriers and needs to work-focused care.

Therefore, the aim of this qualitative study was twofold: 1) to investigate how generalist PTs, OTs and ETs provide work-focused healthcare and 2) to obtain insight into their perceived barriers and needs that affect their ability to address occupational factors.

## Methods

### Design and procedures

This exploratory qualitative study used focus groups to investigate the experiences, barriers and needs that ETs, OTs and PTs face with regard to integrating occupational factors into their treatment. The use of focus groups in exploratory designs has proven to be valuable because of interactivity between participants with regard to generating ideas and evaluating details of experiences and reasons behind attitudes, assumptions and perceptions [15]. Focus groups enable a natural control of data collection because participants interact with each other about certain ideas, assumptions or attitudes [15]. For this study, we organised three focus groups with a mix of generalist PTs, OTs and ETs in the Dutch cities of Nijmegen and Amersfoort between January and May 2019.

The Research Ethics Committee of Radboud university medical center has declared (declaration no. 2018–4465) that this study is not subject to the restrictions in the Dutch Medical Research Involving Human Subjects Act (WMO) and can be carried out (in the Netherlands) in compliance with the Declaration of Helsinki on Ethical Principles for Medical Research Involving Human Subjects.

### Recruitment and participants

Between November 2018 and April 2019, we recruited participants by means of appeals in newsletters from the Royal Dutch Society for Physical Therapy, the Dutch Association of Occupational Therapy and the Dutch Association of Exercise Therapists Cesar and Mensendieck. In addition, therapists were recruited through the professional networks of the authors and the project’s advisory group.

Generalist PTs, OTs and ETs were eligible to participate if they treated working patients with MSDs. However, therapists with additional professional education leading to a specialisation in occupation health were excluded. These criteria were described in the appeals. In addition, the appeals included information about the project, its goals and the focus groups’ procedure. Because a limited number of therapists were interested in participating, it was not possible to use a purposive sampling method; therefore, we included a convenience sample. When possible, we purposively distributed participants between the focus groups to encourage interaction between the three types of therapists. All the study participants agreed to take part in a focus group.

All participants filled in an informed consent form and a questionnaire about demographics, and they all agreed to audio recording prior to the start of the focus group session. Each participant received a gift of €50 for contributing to the focus group.

### Focus groups

We developed a semi-structured interview guide with open-ended questions to stimulate consistency in the questions asked across the groups (see Appendix 1) [15]. The questions were based on the results of previous research [13, 14] and the authors’ expertise. The questions were pilot tested in the first focus group. Because no changes were necessary, the same interview guide was used in all the focus groups. We chose to conduct three focus groups because previous research has found that about 80% of all themes are discoverable within two to three focus groups [16].

Two moderators (WO, TR/PB) facilitated each focus group using a standardised script, and all the groups were audio recorded. All three moderators had experience with conducting qualitative research and one of them (WO) had been trained to conduct qualitative research. The moderators’ work includes roles like researcher (WO, TR), educator (WO), managing director (PB) and policy adviser (TR). They have backgrounds in physiotherapy (WO), social sciences (PB) and movement sciences (TR).

Both moderators in a focus group took notes when needed. No people other than the moderators and participants were present at the focus groups.

Each focus group started with an introduction of that session’s participants in which they were asked about their experiences and reasons for participating in this study. The moderators ensured that all participants contributed to the discussion. Moreover, during the focus group, participants filled in individual questionnaires about their needs (including skills and tools). These contributions were discussed in the groups to calibrate individual ideas and thoughts.

Each focus group lasted about 120 minutes. After each session, the moderators discussed the outcomes and group dynamics and made a summary of the results. Two of the moderators (WO, TR) knew three of the 16 participants. The participants were given no information about the moderators.

### Data analysis

We analysed the data using inductive thematic analysis [17], a method that can be used to identify, analyse and report themes within qualitative data. The audio recordings were transcribed verbatim by two student assistants. All transcripts were analysed by two authors (WO, IU), one of whom (IU) has a background in occupational therapy. Analysis was performed via the following steps: 1) becoming familiar with the data, 2) generating initial codes, 3) searching for themes, 4) reviewing themes, 5) defining and naming main themes, and 6) producing the report [17].

The analysing authors read the transcripts multiple times and generated initial codes with an open-coding system. The codes that initially emerged from the data were compared and discussed until consensus was reached. When no new insights emerged during analyses of the third focus group, data saturation was reached and no additional focus groups were required. After analysing all the data and generated initial codes, the authors sorted the codes into themes based on how the codes are related and linked. This analysis was randomly checked by another author (NH), who is experienced and trained in conducting qualitative research and has a background in physiotherapy.

A summary of the results was emailed to all the focus group participants. They were asked to screen for misinterpretations and to provide additions within 14 days, if needed. The Atlas.ti (version 8.4.24) program was used for coding and managing the analysis, and the research group discussed the supporting quotes related to each theme.

## Results

Sixteen therapists participated in this study, distributed over three focus groups (n=5, 8 and 3). Of the 16 participants, nine were PTs, four were ETs and three were OTs. Given the participants’ availability, it was not possible to include OTs in the second group or ETs in the third focus group.

The participants’ characteristics are presented in Table 1. The mean age of the participants was 44 years (range 25-59) and mean years of experience as a therapist was 19 (range 0.5-37). Qualitative data analyses identified the following themes: Addressing work participation is important, but challenging, the patient's request for help, talking about work, evaluation of work activities, supporting patients, and cooperation with occupational health professionals. An overview of the themes identified is presented in Appendix 2. The presented data are based on the participants’ opinions/statements. Each theme is discussed below, with supporting quotes.

**Table 1:** Demographic profile of the participants

Participant ID	Gender	Age (years)	Profession	Experience (years)	Focus group
1	Female	54	OT	32	1
2	Female	49	ET	26	1
3	Male	41	PT	18	1
4	Female	53	ET	29	1
5	Female	56	ET	35	1
6	Female	59	PT	37	2
7	Male	55	PT	33	2
8	Female	32	PT	8	2
9	Female	34	PT	12	2
10	Female	46	PT	10	2
11	Female	46	ET	20	2
12	Male	25	PT	4	2
13	Female	39	PT	5	2
14	Male	31	OT	0.5	3
15	Female	49	OT	27	3
16	Male	31	PT	9	3

*ET* exercise therapist, *OT* occupational therapist, *PT* physiotherapist

### Addressing work participation is important, but challenging

All participants were aware of the importance of considering occupational factors. OTs mentioned that work is part of their education, and they use questions about their patients’ work as part of getting to know their patient and identifying treatment parameters. OTs and ETs said that, for them, it is natural to take these factors into account because they were taught to focus their interventions on activities of daily life and participation. OTs stated that ‘occupational’ is part of their job title, so their profession is focused on work. One participant explained that work is an important activity for many people:

*‘We call it meaningful activities. Work is definitely one of the meaningful activities for a lot of people […]. I think, for many people, work is a very important aspect of daily life in which they want to maintain participation. It gives them the feeling of being part of society.’ (participant 14 – OT, focus group (FG) 3)*

Another participant stated that, nevertheless, OTs often do not focus on the patient’s work:

*‘If there is one profession which is focusing on making daily activities possible, it is the OT. And, at the same time, the OT frequently misses the opportunity to address work.’ (participant 1 – OT, FG 1)*

Some participants mentioned that if therapists treat from a vision of tailored care, every individual patient needs an individual plan of action, and the therapist automatically addresses occupational factors if these are relevant. Then return to work can become a treatment goal. The participants noted that it is important to have a positive attitude towards cooperation with the patient and to embrace shared decision making in which the patient and therapist share the responsibility to address relevant factors and identify treatment goals.

For PTs, whether they take occupational factors into account depends on their personal interest in occupational health or the environment in which they work. Some participants work in industrial areas where a group of patients presents with the same type of complaints or works for the same employer. Therapists then try to connect these factors and search for similar causes of complaints which, in these cases, are often found in occupational factors. A physical therapy consult lasts approximately 30 minutes, while OTs and ETs often have one hour for a consult. Some of the PTs were concerned that they did not have enough time to address work participation.

Some participants see occupational factors as a possible cause of delay in recovery or the cause of recurrent complaints. Therapists consider occupational factors if they can link these to psychosocial factors – which is a commonly addressed topic in patient interviews. Most participants stated that determining whether there is a link between the patient’s work and the complaints depends on the kind of complaints and is often based on intuition. One participant explained:

*‘Whether I focus on occupational factors in the patient interview depends on the nature of the complaints. For example, if the complaints are related to stress, I will ask much more about work.’ (participant 13 – PT, FG 2)*

In general, participants mentioned a need to shift from a biomedical point of view towards the perspective of the bio-psychosocial model. When a therapist examines the health problem from a more holistic point of view, they will probably see more and other factors, like occupational and psychosocial factors. Some of these factors could be outside the therapists’ own expertise. Therefore, they need to have a positive attitude towards cooperation with specialised therapists or other healthcare professionals, and they need to know their own boundaries of expertise. One participant said:

*‘Be aware of your own limitations. Sometimes it is too complex to treat the patient by yourself. You can’t change that, but you can look at what you can do for your patient.’ (participant 2 – ET, FG 1)*

All participants acknowledged the importance of addressing occupational factors in their treatment. In addition to knowledge about laws and legislation related to work and absenteeism, they agreed that some other skills were necessary to be able to address these topics. Most of them are basic skills of the therapist, like building trust. Asking more about work can be a stimulating factor for building connection, getting to know the patient better and building a trusting relationship. Participants noted that effective communication skills are important and they mentioned several tools that can be used to enhance communication. For instance, some participants mentioned that motivational interviewing is a valuable method. The goal of communication is to empower patients and help them gain insight in their own situation and the possibility that their work causes or contributes to their complaints. Other important goals of communication that were mentioned include facilitating behaviour change related to work posture, pausing behaviour, maintaining work-life balance, and communicating with the employer or occupational healthcare professional.

In the Netherlands, patients can visit a PT, ET or OT without a referral (direct access). Some participants wondered whether getting a referral from a medical specialist could contribute to increasing attention on occupational factors. However, two therapists who often work with a referral did not have the experience that doctors pay attention to their patients’ work. One of them explained:

*‘I always treat my patient based on a referral. The complaints always influence the ability to work, but that is never mentioned in the referral.’ (participant 1 – OT, FG 1)*

Although all participants had a bachelor’s degree and most were more specialised in specific treatments, they saw a general need for better communication skills in order to facilitate behaviour change and self-management in their patients.

Another identified need with regard to addressing work participation is having a kind of ‘sparring partner’ with whom to discuss work-related factors, legislation and regulations, and advice about workload. However, the participants were not yet clear who that professional could be. One participant also suggested having a forum:

*‘A kind of forum where you can publish a case. But I wonder who would maintain such a site.’ (participant 1 – OT, FG 1)*

#### The patient's request for help

Most participants said that whether they focus on occupational factors largely depends on whether patients mention their work themselves. All participants mentioned that an important reason for addressing work participation is its identification in the patient’s request for help in the patient interview. Therapists focus on that request for help because they assume that it is an important factor and goal for the patient. One participant said:

*‘Well, when patients come to me with a request for help linked to their work or work participation, it’s obvious that it’s important to them. However, I assume that work is important for most people.’ (participant 9 – PT, FG 2)*

However, participants mentioned that therapists need to be aware of the real message and request for help, which is not always expressed verbally. Therapists need to work with the patient to explore what really matters and what their treatment goals will be. If patients were more aware of the possible relationship between their complaints and their work, they would be more likely to express this to the therapist and, subsequently, the therapist would be more likely to address it. Some participants stated that asking a patient to fill in a questionnaire about occupational factors or overall participation prior to the first consult could help to make them more aware of the relevance of their work. One participant explained:

*‘I think it would be helpful if you could prepare patients in advance with certain questions. If you know that your patient has a job, you could give them some questionnaires about their work prior to the first consult […]. Then they would know to expect questions about it.’ (participant 15 - OT, FG 3)*

Therapists want to support their patients in focusing on these factors and they want their patients to be more aware of this topic. Participants mentioned that they are aware that the occupational healthcare and social security system is perceived as complex, and the patients often do not know what their rights and duties are.

Participants also highlighted that patients need self-management skills to manage the period in which they are absent from work, as well as skills to change their (movement) behaviour. Patients need to take the lead and become the managers of their own recovery. Because of privacy legislation, there is little or no communication between therapists and employers. Instead, patients decide which health-related data to share with their employer.

### Talking about work

Some participants take a ‘reflective’ role in dialogue with clients and talk about the extent to which work plays a role in the development or persistence of their complaints. Most participants analyse which work-related factors could play a role in increasing or decreasing their patients’ complaints and advise patients about their conclusions. One participant reflected:

*‘I just start the conversation with people and try to find out whether there is a connection between their work and their complaints […]. I use mirroring to help my patients get some insight into what the cause of the problem could be and what they can do about it.’ (participant 9 – PT, FG 2)*

All participants agreed that it is important to be aware of the meaning of work for the patient. That is not only financial, but is literally a big part of their life. For some people, work provides a daily structure they can hold onto, and for others the social aspect of meeting colleagues is very important and can also contribute to good health. One participant said:

*‘I think work also gives a kind of meaning to life. Work is important. Some people see their colleagues more often than their own partner. It is a significant part of your life, your day.’ (participant 3 – PT, FG 1)*

However, work can be a factor that negatively influences recovery. One participant explained:

*‘I look for factors that contribute to the persistence of complaints. Then you automatically also need to address occupational factors.’ (participant 4 – ET, FG 1)*

It is normal for ETs and OTs to ask about these factors. PTs often address these factors in later consultations, especially when patients do not recover as quickly as expected. Some PTs and ETs automatically take work into account because they ask about work postures and movement/activities. To create awareness, some participants ask patients whether they see a relationship between their complaints and their work. One participant reflected:

*‘During the patient interview, I sometimes ask questions about work, although I know that I will not get an accurate answer. I ask these questions to raise awareness and make patients start thinking about the connection between their complaints and their work activities.’ (participant 16 – PT, FG 3)*

Determining work relevance during history taking is mainly based on gut feelings or on what the patient indicates in their request for help. Most participants indicated that they talk with the patient and investigate the extent to which there is a relationship between the patient’s complaints and their work. Participants who ask patients about topics like the kind of work they do also ask about specific work characteristics and ask patients if they think there is some kind of causal connection between their complaints and their work. They also ask about daily living activities and what is important to the patient, while assuming that work is a big part of the patient’s life.

During patient interviews, some participants try to discover whether there is any connection between the patient’s complaints and work activities. That helps therapists choose a treatment that fits the patient’s need, in close collaboration with the patient, which facilitates the patient’s being in control. One participant pointed out that adapting therapy to the patient’s needs is essential:

*‘Sharing knowledge, specific knowledge and experience, but with a focus on that request for help: that is what people are motivated to work for, not something that I want as a therapist.’ (participant 4 – ET, FG 1)*

Some participants said that they ask about the type of work and which activities are performed with what frequency and duration. They often ask: ‘What does your normal day look like?’ OTs ask this question with the goals of getting to know the patient better and forming an idea of the possibilities for influencing and controlling their work.

One of the OTs carries out workplace investigations but is also interested in the activities patients perform in their spare time. Some of the OTs try to differentiate between complaints caused or aggravated by work and by leisure time activities. Most of the OTs use the Canadian Occupational Performance Measurement (COPM), a dialogue tool that prompts them to ask questions about all aspects of health and participation. One participant explained:

*‘The patient describes an average day. After that, you can explore which activities are problematic and how the patient prioritises these activities. You score the prioritised problems, which provides insight and facilitates the setting of treatment goals.’ (participant 15 – OT, FG 3)*

One participant mentioned the need for another tool:

*‘I’m sometimes looking at the extent to which occupational factors influence recovery or are the cause of complaints. Can I filter out the patients for whom occupational factors play a major role? I need a kind of tool for that.’ (participant 16 – PT, FG 3)*

#### Evaluation of work activities

After asking questions in the patient interview, some therapists also ask the patient to show some work activities. Some participants said as therapists not specialised in occupational health, they often find it difficult to get a clear view of what kind of work activities a patient does and how they are performed. Some participants have experience with workplace investigations or have a colleague who conducts workplace investigations. Sometimes it is obvious that the workplace is not designed or used correctly. However, most participants emphasised that they need more knowledge about work activities, the work environment, self-regulation and workplace ergonomics to perform a decent work-focused physical examination.

One OT uses workplace investigations to get a complete picture of the patient and often sees that the problem is not only related to work at the workplace, but it is also related to continuing work at home because of deadlines and a high workload. Most participants expressed the need to make a good analysis of the risks posed by the patient’s work and the influence of work on their recovery, with the aim of treating the patient adequately. A few participants ask patients to film themselves at their jobs.

Two participants ask patients to perform some of their work activities. This can provide valuable insights, but sometimes patients show the desired way of performing these activities rather than what they actually do:

*‘If you ask your patient to perform an activity, they often show how they think they should do the activity, instead of how they actually perform the activity at work. So I ask them to perform the activity as they think it has to be done and, after that, show the activity again using the way you do it in your routine.’ (participant 12 – PT, FG 2)*

### Supporting patients

All participants mentioned a need for good communication skills to begin work-focused treatment and guidance and to support self-management and behaviour change. Furthermore, participants want to give their patients advice about how to move healthily and how to balance their workload and private life.

Paying more attention to work-related complaints also requires the creativity to convert work actions into exercises. One participant said:

*‘Sometimes I use exercises, or I observe how my patient moves while doing his job. I try to convert that into practical interventions.’ (participant 13 – PT, FG 2)*

The majority of participants agreed that therapists must have basic knowledge, especially about employment laws and regulations. For example, if a therapist advises a patient to (temporarily) work fewer hours, the therapist should be aware that this not only affects the complaints, but it can also affect the patient’s income and financial situation. Participants doubt that they are allowed to advise patients to stop performing activities that are problematic to perform due to the complaints. Almost all participants struggle with determining the extent to which they are responsible in the process of their patient staying at work or returning to work. Given the presence of different stakeholders with different interests, the social security system and the financial consequences, therapists perceive this as complex. Contrary to advising patients to (temporarily) stop participating in sports, advice about work has more radical consequences. Thus, the insecurity remains: what are a therapist’s rights and obligations with regard to advising a patient to work or not to work? Does a therapist need to know all the possible financial consequences and be able to advise the patient about whether to stop working? One participant mentioned:

*‘The number one question from patients is, “Do I need to ask for sick leave?” That is one of the most difficult questions for me, and I answer that it is not within my authority to advise you about that.’ (participant 11 – ET, FG 2)*

Therefore, some participants advise their patients to ask their employer or occupational health physician for advice, while other therapists send patients back to the general practitioner (GP).

A few other participants give patients advice about whether to perform work activities. Sometimes, that advice is about stopping work because the therapist is worried that work activities are worsening complaints. One participant expressed:

*‘Well, one person says you have to do this and that, but it seems quite logical that someone who just had surgery on his shoulder can’t lift and carry what he regularly does: plates that weigh 15 kg. However, he still gets the advice to go to work.’ (participant 7 – PT, FG 2)*

Most participants mentioned that they want to have more insight and knowledge about the treatment possibilities as well as the patient’s ability to work despite complaints (thinking in possibilities). One participant said:

*‘Especially in people with chronic pain, work can be difficult. They [patients] often say that they cannot work, but there are always possibilities. Then I try to find solutions.’ (participant 11 – ET, FG 2)*

The majority of participants want additional knowledge about laws and regulations related to work and absenteeism and want to know where they can find more specific information about workload and other occupational factors.

### Cooperation with occupational health professionals

All participants had an urgent need for more cooperation with other healthcare professionals. However, only three participants were aware of the existence of PTs, OTs or ETs who are specialised in occupational health. After receiving information about the expertise of therapists specialised in occupational health in the focus group, participants thought consulting these therapists and referring patients to them would increase the quality of care.

The added value of these specialised therapists is not clear. This means that referrals do not take place. Some participants stated that they want to collaborate, but they do not know who the important stakeholders are, how to find them, and which one would be the best to refer to. One participant elaborated on why he is not working together with health professionals specialised in occupational health:

*‘OK, if I have no clue about what other disciplines do in their job, how can I actually know when and to whom I should refer patients […]?’ (participant 16 – PT, FG 3)*

Participants experience a lack of knowledge about laws and regulations and about the tasks and responsibilities of occupational healthcare professionals. One participant said:

*‘You do not pay a lot of attention to social security because you are focused on helping the patient to get better physically. Well, probably it is not our responsibility, but nevertheless you need to know where you can refer your patient.’ (participant 5 – ET, FG 1)*

Participants who already work together and have positive experiences with other occupational healthcare providers often see their added value and realise that no one can know everything. One participant remarked:

*‘It is such a complex situation. In that way, it’s strange that we think we can do it alone.’ (participant 1 – OT, FG 1)*

Possible conflicts of interest are perceived as a barrier to cooperation with other occupational health professionals. The patient is the therapist’s client and sometimes the therapist has the impression that occupational health professionals and the patient’s employer have different priorities. Some participants mentioned situations in which their patient had the impression that an occupational health physician was acting in the interest of the employer and the patient felt pressured to return to work quickly. Some participants had experienced that an occupational health physician did not take their expertise seriously and did not follow the advice they gave.

Furthermore, the Netherlands has strict laws about protecting privacy. Therapists are not allowed to communicate a patient’s private or personal data without the patient’s consent. Generally, participants feel that the law limits their ability to communicate with other stakeholders about a patient’s situation. They would rather advise their patient to communicate with the occupational health physician and their manager/employer than to contact them directly. One participant explained that there is sometimes contact between the therapist and employer, if the employee wants that:

*‘Only if the employer asks for contact, not the other way around [...]. If there is a request, I ask the employee to tell his manager that he can contact us.’ (participant 1 – OT, FG 1)*

After the focus group moderators offered a brief explanation about the expertise of specialised therapists, the participants agreed that specialised therapists could add value for patients with work-related complaints. A few participants already experienced the added value in practice because they already work together with other healthcare professionals in rehabilitation centres or multidisciplinary healthcare centres. When the therapists are direct colleagues or work together in the same network, this facilitates cooperation.

Having more knowledge about each other’s expertise and having an easy way to find each other and communicate with each other are positive factors that support collaboration. One participant had a good experience with an occupational health physician:

*‘We have an occupational health physician who contacted us to cooperate, so he is able to refer employees faster and easier […]. He contacted us because we have a lot of expertise in our practice and can act quickly if treatment is necessary.’ (participant 3 – PT, FG 1)*

A lack of opportunities to communicate is an important reason why there seems to be a cooperation gap between regular healthcare and occupational healthcare professionals and the employer. The laws and regulations about privacy and about tasks and responsibilities give most of the participants a feeling that the situation is complex. They would rather withdraw than take a proactive or coordinating role. If they do not feel capable enough to coach a patient to stay at or return to work, they refer the patient back to the GP. Therapists are not familiar with communicating with occupational health physicians or employers. Moreover, occupational health physicians are often difficult to contact. One participant often works with the patient, employer and occupational health physician in return-to-work interventions. She developed a protocol for regular healthcare for patients with chronic pain. She explained:

*‘We struggled a long time; each stakeholder had their own ideas, occupational health physicians were not easy to contact and we experienced a difficult collaboration with the employer. We focused on how we can work together to make a best-practice approach together.’ (participant 11 – ET, FG 2)*

The majority of the participants took part in the focus groups because they saw a need for more cooperation with other healthcare professionals and a simultaneous lack of knowledge about the tasks and added value of other healthcare professionals, more specifically occupational healthcare professionals. One participant said:

*‘I can only speak for myself, but I think there is much to gain from interprofessional cooperation. What is our added value to each other?’ (participant 14 – OT, FG 3)*

The participants expressed a strong need to define boundaries and responsibilities between generalist therapists and specialised therapists. The more insight the therapists have into what someone else can add, the better they can refer. Therapists need to know when and to whom they should refer and must be able to find those professionals. Participants expressed a need for more knowledge about what they are supposed to do and when they should refer a patient to a specialised therapist or other occupational healthcare professional. Most participants wish they had a decision aid to use in deciding when to collaborate with a specialised therapist or other occupational healthcare provider. One participant specified:

*‘I need some kind of screening tool to decide if and when there is an indication to refer the patient. I agree that it [working together with different professionals] is complementary and of added value, but at the same time I don’t know what the added value is or can be.’ (participant 16 – PT, FG 3)*

## Discussion

This study investigated how generalist PTs, OTs and ETs provide work-focused healthcare and which barriers and needs they experience when addressing occupational factors. Participants were aware of the importance of taking occupational factors into account. Whether they address occupational factors is largely dependent on the patient’s request for help. One important finding is that all the participants felt an urgent need for more cooperation with other healthcare professionals, and most of them lacked knowledge about therapists specialised in occupational health.

Several other needs were identified in this study. These included knowledge about laws and legislation and skills for identifying and addressing work-related or work-relevant complaints (e.g. knowledge about work activities, the work environment, self-regulation and workplace ergonomics). With regard to knowledge about laws and regulations, participants felt uncertain and doubted whether they are allowed to advise a patient to stop performing activities that are problematic due to the complaints. Almost all participants struggle with determining the extent to which they are responsible for whether their patients stay at or return to work. Moreover, participants largely did not know their rights and obligations with regard to advising patients about work participation and absenteeism, and they perceived such matters as complex.

Therapists also need skills for making a good analysis of the risks of the patient’s work and the influence of work on their recovery. As mentioned before, participants also needed more knowledge about therapists specialised in occupational health and other occupational healthcare providers. Moreover, they wanted a decision tool for referral and a tool to explore the extent to which occupational factors influence recovery or cause the complaints.

Some of these needs were also found in a previous study in which the most frequently mentioned need of PTs were the ability to bill for a workplace assessment (60%), questionnaires about the patient’s work participation (53%), screening lists to assess the extent to which the patient’s complaint is work-related (52%), more knowledge about occupational-health-related laws and regulations (50%) and more knowledge about the domain and position of the physiotherapist specialised in occupational health (43%) [13]. Those findings indicate that these needs are also relevant to at least some ETs and OTs. In the current study, most participants also expressed a need for better communication skills in order to facilitate behaviour change and self-management in their patients.

It seems that some of the results from this study are also applicable to other healthcare professionals. Previous qualitative research showed that although GPs find it important to pay attention to work during their consultations, they need more knowledge and communication skills and better cooperation with occupational physicians to manage work-related problems. These GPs feel that they lack the knowledge to advise patients specifically about their working environment and the GPs experience a lack of access to and communication with occupational health physicians [18].

Our study included a mix of PTs, OTs and ETs. Although the majority of participants had a background in physiotherapy, we obtained some insight into the differences between the professions. We found that ETs and OTs consider it normal to ask about occupational factors during the diagnostic process, while PTs often address this in later consultations, especially in cases of delayed recovery. Moreover, whether PTs take occupational factors into account depends on their personal interest in occupational health, the environment in which they work or their specialisation. Although OTs mentioned that work is part of their education, they often do not focus on the patient’s work. The results from all three professions suggest that providing work-focused care could be improved.

Almost all participants were unaware of the existence of PTs, OTs or ETs who are specialised in occupational health. For example, PTs specialised in occupational health promote healthy work, strive to prevent injuries and illnesses, and manage work-related conditions [9]. Previous studies also found a lack of cooperation between generalist PTs and PTs specialised in occupational health [13, 14]. For example, of the physiotherapists who had no direct colleague who was specialised in occupational health, about 87% never or rarely referred a patient to a PT specialised in occupational health [13]. Previous research also found a lack of knowledge about the added value of PTs specialised in occupational health and the differences between their work and regular physiotherapy [13, 14]. Our study also found such a lack.

Moreover, almost all our participants struggled with when to refer a patient to other (occupational) healthcare professionals, and they often only consulted a specialised therapist or referred a patient to a therapist specialised in occupational health when recovery was delayed. This could have a negative effect on the patient’s recovery time because addressing occupational factors at an early stage could be beneficial. Participants expected that collaboration would be facilitated by having more knowledge about each other’s expertise, easier ways to find each other and enhanced communication.

It is important to take a multidisciplinary approach that involves therapists who are specialised in occupational health and other occupational healthcare professionals [9]. Such an approach can be especially beneficial when workplace adjustments are needed or aspects of work require change (e.g. if work-related cognitive demands require modification) [9]. In this study, participants perceived that possible conflicts of interest create barriers to collaboration with other occupational health professionals. Moreover, participants felt that privacy laws limit their ability to communicate with other stakeholders about the patient’s situation. They would rather advise their patients to communicate with their occupational health physician and manager/employer than contact them directly. This is contrary to previous research, which found that 72.5% of the PTs had had contact with an occupational health physician [13], although it is unknown how often this contact took place.

Previous research found that a minority of PTs (17.6%) have contact with employers [13], which is supported by the results of this study. However, Sennehed et al. [19, 20] found that a structured workplace dialogue (a physiotherapist-led interview with the patient and an interview with the employer and, finally, a meeting between patient, employer and physiotherapist) in a randomised controlled trial among patients with acute and sub-acute low back and neck pain was cost effective and improved work ability [19, 20], which indicates the importance of a dialogue with the employer and highlights a potential role for PTs. However, this could be difficult to implement because of a lack of funding.

In general, participants mentioned that it is necessary to make a shift from a biomedical point of view towards the perspective of the bio-psychosocial model. This is supported by many publications [10]. Some PTs and ETs automatically take work into account because they ask about work postures and movement/activities, but this could also be a sign of a biomedical focus. Participants in this study expected that looking at a health problem from a more holistic point of view would facilitate looking at occupational and psychosocial factors. However, many studies have found that PTs address psychosocial components in a suboptimal way [22,25,26,27,28,29,30,31]. Previous work also found that the treatment orientation beliefs held by PTs influence their clinical practice and the advice they give to patients [22]. Therapists with a greater biomedical orientation and fear avoidance beliefs about chronic low back pain were found to be associated with the advice to restrict return-to-work duties, a higher perception of risk associated with work or activity, and increased certification of sick leave [22].

The patient’s role is also important to mention, because most of our study’s participants said that whether they focus on occupational factors largely depends on whether patients mention their work themselves or whether they identified work as the patient’s request for help in the patient interview. However, as some participants noted, patients are not always aware of the importance of their work (or work situation). A previous study also found that when patients are unaware of the relationship between their disorder and their work, it can be difficult to discuss work and work-related factors [14]. In the current study, participants wanted to support their patients in focusing on these factors and wanted their patients to be more aware of this topic, which could be facilitated by a pre-treatment questionnaire.

Of course, the patient’s perspective is also important. Therefore, we also conducted focus groups with patients to investigate their perspectives. The results of these focus groups will be published elsewhere.

Participants also highlighted that patients need self-management skills to manage their complaints at work or during the period in which they are absent from work, as well as skills to change their (movement) behaviour. Patients need to take the lead and become the managers of their own recovery. Previous work recommends self-management and providing self-management support [10], however, the way PTs, OTs and ETs address self-management was found to be suboptimal and could be improved [31,35,36].

### Strengths and limitations

This is the first study to investigate how generalist PTs, as well as OTs and ETs, provide work-focused healthcare. This study largely reinforces the results found in previous studies on PTs [13, 14] and provides preliminary evidence for the idea that their experiences and needs do not differ greatly from those of ETs and OTs, although some differences exist.

Strengths of this study include its strong qualitative design: we conducted three focus groups, two independent researchers performed data analysis using inductive thematic analysis [17] and results were confirmed by participants. The sample used in this study represents therapists with various ages and years of work experience. This study also fulfilled all criteria mentioned in the consolidated criteria for reporting qualitative research (COREQ), a 32-item checklist for interviews and focus groups [37].

This study also has some limitations. Although participants were distributed over three focus groups, the number of participants in each group was small and inconsistent (e.g. the last focus group consisted of only three therapists). It was also impossible to include a mix of PTs, ETs and OTs in all three focus groups. A disadvantage of small focus groups is that they limit the total range of experiences [15]. However, by including 16 participants and reaching data saturation, we believe we have collected sufficient in-depth ideas and experiences from the participants.

Due to the limited number of people willing to participate, we had to use a convenience sampling technique. This could have led to selection bias because people with an interest in occupational health could be more willing to participate. We also recruited participants using newsletters and the networks of the authors and the project’s advisory group.

It is unknown if the results of this study are transferable to other countries, because differences in (occupational) healthcare systems exist [9]. Moreover, in many countries for example, no specialisation in occupational health physiotherapy exists [9], which might limit intra-professional cooperation.

As the researchers who conducted this study were involved in all the different phases of the study, researcher bias may have affected the results of this study. Using transparent reporting and reflective awareness, including taking into account our assumptions, taking field notes, multiple coders of the data, participant checking, and discussion between the researchers, we tried to avoid bias [37, 38].

## Clinical implications and future research

This study and previous studies [1, 2] found that although therapists think it is important to address the patient’s work and occupational factors, they generally do not sufficiently integrate this knowledge into their practice. PTs, OTs or ETs lack specific knowledge, including knowledge about occupational healthcare providers (e.g. PTs, OTs or ETs specialised in occupational health). Therefore, we recommend that practitioners pay attention and better integrate their patient’s work into their practice and improve their knowledge and skills. We also recommend that PTs, OTs and ETs work together with occupational healthcare providers, especially with PTs, OTs and ETs specialised in occupational health. Additional measures to improve this are needed. Moreover, research investigating the way therapists address work participation in other countries is recommended.

Because most of the participants in this study mentioned that whether they focus on occupational factors largely depends on whether patients mention their work or if this is identified as the patient’s request for help in the patient interview, it will be important to conduct further research to investigate patients’ ideas and needs with regard to addressing their work. Moreover, we recommend that materials be developed to support patients’ awareness of the importance of addressing their work participation.

## Conclusions

Participants in this qualitative study were aware of the importance of taking occupational factors into account. However, how PTs, OTs and ETs address work participation and the extent to which they do so can be improved. Although ETs and OTs consider it normal to ask patients about occupational factors, the provision of work-focused care could be improved in all three professions. There was a lack of knowledge about and cooperation with occupational health professionals, including PTs, OTs or ETs specialised in occupational health. Participants needed more knowledge about therapists specialised in occupational health and other occupational healthcare providers. Improving cooperation between PTs, OTs and ETs and occupational health professionals, including PTs, OTs or ETs specialised in occupational health is important.

## Supplementary Information

**Additional file 1: Appendix 1.** Interview guide (translated)

**Additional file 2: Appendix 2.** Overview of themes and sub-themes

## Data Availability

The datasets generated and/or analysed during the current study are not publicly available due to the privacy of the participants, but are available from the corresponding author on reasonable request.

## Abbreviations

MSDs: Musculoskeletal disorders
PTs: Physiotherapists
OTs: Occupational therapists
ETs: Exercise therapists
WMO: Dutch Medical Research Involving Human Subjects Act
COREQ: Consolidated criteria for reporting qualitative research
GP: General practitioner
FG: Focus group
