# Supplementary material for: Therapists’ experiences and needs with regard to providing work-focused care: a focus group study
Source: BMC Musculoskelet Disord. 2021 Nov 2;22:923. doi: 10.1186/s12891-021-04806-4 (PMC8565033; doi:10.1186/s12891-021-04806-4)
Supplement: Supplementary file 1 — Additional file 1: Appendix 1. Interview guide (translated) [file 12891_2021_4806_MOESM1_ESM.docx]

**Appendix 1** Interview guide (translated)

**Introduction**

- The moderator gives a short introduction to the project and some background information
- The moderator gives a short introduction about participating in a focus group
- The participants introduce themselves (name, specialisation, experience)

**Addressing the patient’s work**

- How do you address your patient’s work?
- Why do (or don’t) you think addressing the patient’s work has added value?
- What are your experiences with working together with other (occupational) healthcare providers?

***Break***

**Work-focused care**

- Exercise: Participants fill in individualised questions about needs (including skills and tools)
- Plenary discussion about previous exercise

**Therapists specialised in occupational health**

- Do you refer patients to and/or work together with therapists specialised in occupational health? Why or why not? *(If needed the moderator will provide a brief explanation about the expertise of specialised therapists).*
- Do you refer patients to and/or work together with other occupational healthcare providers? Why or why not?

**Closing**

- What do you think was the most important topic addressed in this session?
- Did we miss any topics about the subject of this session?
