# Supplementary figures and images for: Therapists’ experiences and needs with regard to providing work-focused care: a focus group study
Source: BMC Musculoskelet Disord. 2021 Nov 2;22:923. doi: 10.1186/s12891-021-04806-4 (PMC8565033; doi:10.1186/s12891-021-04806-4)

**Appendix 2** Overview of themes and sub-themes


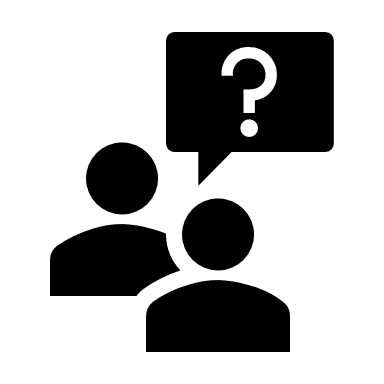

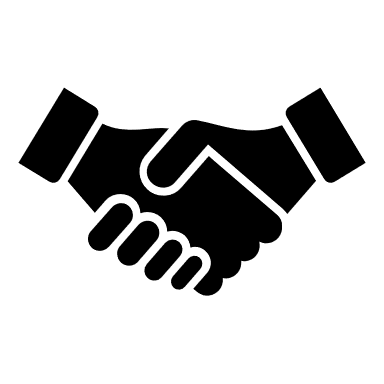

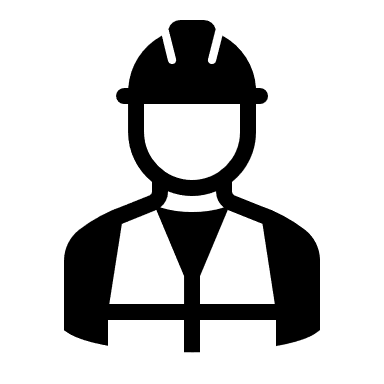

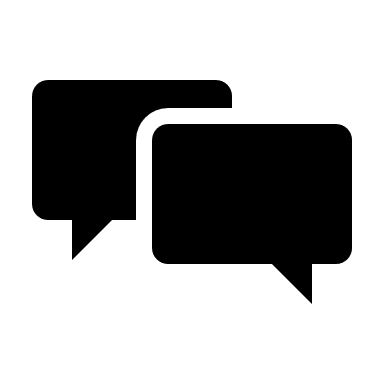

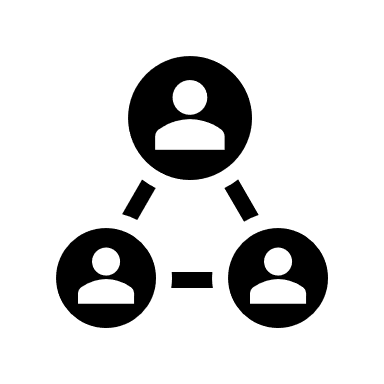
**
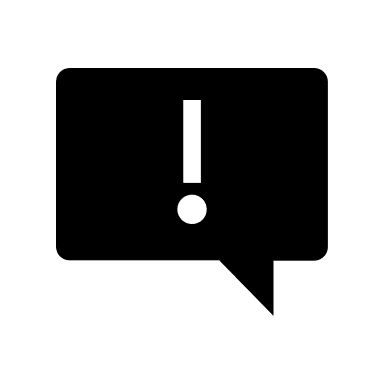
**

Supplement: Supplementary file 2 — Additional file 2: Appendix 2. Overview of themes and sub-themes [file 12891_2021_4806_MOESM2_ESM.docx]
